# Development and Validation of a Disease-Specific Oromandibular Dystonia Rating Scale (OMDRS)

**DOI:** 10.3389/fneur.2020.583177

**Published:** 2020-11-03

**Authors:** Kazuya Yoshida

**Affiliations:** Department of Oral and Maxillofacial Surgery, National Hospital Organization, Kyoto Medical Center, Kyoto, Japan

**Keywords:** oromandibular dystonia, rating scale, validity, reliability, quality of life, disease severity, Toronto Western Spasmodic Torticollis Rating Scale-2, Cervical Dystonia Impact Profile-58

## Abstract

**Background:** Oromandibular dystonia manifests with sustained or task-specific contractions of the masticatory, tongue, and/or other muscles in the stomatognathic system. Since its symptoms can vary, it has been difficult to objectively measure disease severity and post-treatment changes.

**Objective:** To develop and validate a comprehensive measurement tool for oromandibular dystonia.

**Methods:** An examiner-rated scale included three subscales for severity, disability, and pain, modified specifically for oromandibular dystonia from the Toronto Western Spasmodic Torticollis Rating Scale-2. To evaluate the severity of each subtype of oromandibular dystonia, four of the six items were selected according to the subtype (jaw closing dystonia, tongue dystonia, jaw opening dystonia, jaw deviation [protrusion] dystonia, and lip dystonia). A patient-administered questionnaire based on clinical features and other relevant aspects associated with oromandibular dystonia was developed, which included five subscales: general, eating, speech, cosmetic, and social/family life. The questionnaire, examiner-rated scale, and four subscales (sleep, annoyance, mood, and psychosocial functioning) of the Cervical Dystonia Impact Profile-58 were combined to construct the oromandibular dystonia rating scale (OMDRS). The reliability and validity of the scale were assessed using clinimetric testing.

**Results:** Six hundred and eighteen patients with oromandibular dystonia (394 women and 224 men; mean age, 51.7 years) were evaluated by the OMDRS. The overall OMDRS showed high-level internal consistency measured by Cronbach's alpha (0.95) with a logical factor structure. Cronbach's alpha for the subscales was satisfactory to excellent, ranging from 0.72 to 0.94. All items revealed acceptable inter-rater reliability (kappa > 0.4, interclass correlation coefficient > 0.6). Repeated ratings of videotapes revealed acceptable intra-rater reliability for all items (kappa > 0.76, interclass correlation coefficient > 0.86). Test-retest reliability showed a significant (*p* < 0.001) correlation efficiency. The OMDRS showed significant (*p* < 0.001) convergent and discriminant validity and significant (*p* < 0.001) sensitivity to changes after botulinum toxin therapy.

**Conclusion:** The OMDRS can be useful for the comprehensive evaluation of disease severity, disability, psychosocial functioning, and impact on the quality of life as well as therapeutic changes in patients with oromandibular dystonia.

## Introduction

The stomatognathic system is an anatomic and functional unit comprising several structures with hard and soft tissues. The hard tissues include the mandible and maxilla, the dental arches and teeth, and the temporomandibular joint, while the soft tissues are the masticatory muscles, the nervous and vascular supplies, and the salivary glands. The system plays critical roles in various indispensable functions of daily living such as chewing, swallowing, speaking, breathing (maintenance of upper airway patency), and facial expression.

Dystonia is characterized by sustained or intermittent muscle contractions that cause abnormal movements or postures (1). Oromandibular dystonia is a focal dystonia involving the masticatory, lingual, and/or muscles in the stomatognathic system (2–6), which presents as jaw closing, jaw opening, lingual, jaw deviation, or jaw protrusion dystonia, or a combination of these subtypes (2–6). Symptoms related to oromandibular dystonia include masticatory disturbance, limited mouth opening, muscle pain, dysphagia, dysarthria, esthetic problems, and temporomandibular joint dislocation (2–6). Most of these symptoms can result in impaired activities of daily living, social embarrassment, cosmetic disfigurement, and a significant impact on a patient's overall quality of life (6). Upper airway obstruction due to temporomandibular joint dislocation resulting from severe jaw opening dystonia (7) or aspiration pneumonia related to lingual dystonia (8, 9) can be life-threatening in some patients. The symptoms and clinical features may be significantly more variable, critical, and complicated than those observed in other types of focal dystonia. For neurologists, oromandibular dystonia has been regarded as the most challenging dystonia to treat (10).

In 2002, we reported a simple clinical scoring system according to the subscores for pain, mastication, speech, and discomfort and evaluated 44 patients with oromandibular dystonia before and after muscle afferent block therapy (2, 3). In 2010, Merz et al. (11) developed and validated the Oromandibular Dystonia Questionnaire (OMDQ-25); it is the first valid instrument for measuring health-related quality of life in patients with oromandibular dystonia. Recently, an oromandibular screening questionnaire was developed and validated for differential diagnosis (6) using a method for the development of health measurement scales (12) and scales for craniocervical dystonia (11, 13). Several measurement tools have been used to evaluate various types of dystonia; however, only a few instruments have been assessed in the clinimetric context (14, 15). This study aimed to develop and validate a comprehensive disease-specific oromandibular dystonia rating scale.

## Materials and Methods

The oromandibular dystonia rating scale (OMDRS) was developed and confirmed to be reliable and valid based not only on standard methods for health measurement scales (12, 16) but also on methods for creating scales for oromandibular dystonia (11), craniocervical dystonia (13, 17–19), and Parkinson's disease (20–22). The classical test theory comprises a series of principles that allow physicians to determine the efficacy of appropriate proxy indicators for estimating unobservable variables (16). Using the classical test theory (12, 16), the OMDRS was evaluated with respect to internal consistency quantified by Cronbach's alpha (23), reliability, item-to-total correlations, Cronbach's alpha if items were removed, distributional skewness, and potential ceiling or floor effects for each subscale of the OMDRS. Furthermore, construct validity (convergent and discriminant validity) was examined through exploratory factor analysis, sensitivity to change, and inter- and intra-rater reliability.

### Item Generation

Structured interviews were conducted for each patient by the author, who has 30 years of experience in treating patients with oromandibular dystonia at both oral and maxillofacial surgery and neurology departments. Clinical features, symptoms, complaints, medical history, and other relevant issues that were adversely affected by the symptoms were described in detail in the medical records. A list of phenomenological aspects was identified based on the medical records and patient feedback for a preliminary version of the questionnaire with 42 items. Some items that were modified from those of the Craniocervical Dystonia Questionnaire (CDQ-24) (13) and OMDQ-25 (11) were added to the preliminary questionnaire.

Subscales for severity, disability, and pain were specifically modified for oromandibular dystonia from those of the Toronto Western Spasmodic Torticollis Rating Scale-2 (TWSTRS-2) (17). The sleep subscale of the Cervical Dystonia Impact Profile-58 (CDIP-58) (24) was slightly modified for oromandibular dystonia. The examiner-rated scale, preliminary 42-item questionnaire, and four subscales (sleep, annoyance, mood, and psychosocial functioning) of CDIP-58 as a quality of life measure were combined to construct the preliminary OMDRS. Patients were videotaped using a protocol for oromandibular dystonia at the first visit and before and after any treatment (Table 1).

**Table 1:** Video examination protocol for oromandibular dystonia.

1.	At rest (10 s)
2.	Count from 1 to 10 aloud
3.	Open/close mouth (5 times)
4.	Lateral movements (5 times)
5.	Jaw protrusion (5 times)
6.	Tongue protrusion (hold for 5 s)
7.	Hold long vowel: “Ahh.” for 5–10 s
8.	Read: sentences
9.	Gum chewing (30 s)


Using the content validity ratio, each item was assessed by experts in movement disorders to determine whether the item was relevant to the severity rating. After explaining content validity, the raters evaluated each item on a 4-point scale as follows: 4, highly relevant; 3, quite relevant or highly relevant but needs rewording; 2, somewhat relevant; and 1, not relevant (12). The content validity ratio for each item was defined as follows: content validity ratio = (ne - N/2)/(N/2), where ne is the number of raters that indicated the item as relevant with regards to the severity rating for oromandibular dystonia, and N is the total number of raters (12). To ensure that the results are not due to chance, items lower than 0.85 were discarded (12).

### Scale Generation

The preliminary rating scale was administered to 220 consecutive patients (141 women and 79 men; mean age ± standard deviation, 51.5 ± 16.1 years).

**Figure 1:**
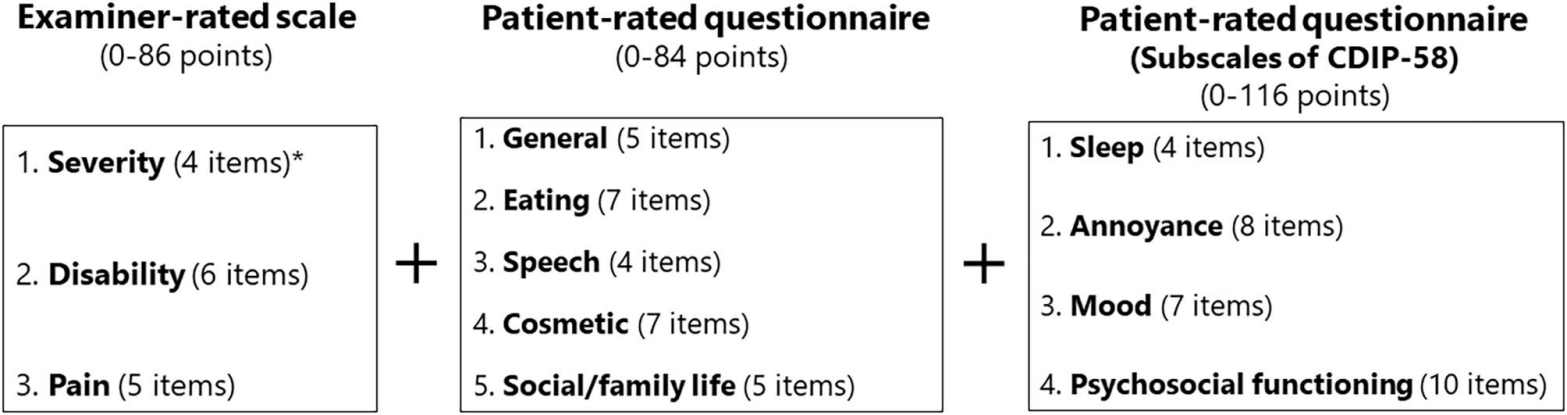
Structure of the oromandibular dystonia rating scale. The asterisk indicates that the subscale of severity was calculated from four of the six items according to the five subtypes of oromandibular dystonia (jaw closing, tongue, jaw opening, jaw deviation [including jaw protrusion], and lip dystonia).

A combination of exploratory factor analysis (principal components method) and cluster analysis was applied for scale generation (12) to determine the most appropriate number of relevant factors. Both orthogonal and oblique rotations were tested to assess the independence of the resultant rotated factors. A minimal factor loading of 0.4 was accepted as a criterion for factor relevance. The criteria for dual loading were set at 0.25. Redundancy was assessed by items loading multiple factors (19). If an item had multiple clinimetric failures, such as poor item-to-total correlation (defined as ≤ 0.3), poor factor loading (defined as ≤ 0.4), or a skewness out of the range of −1.50 to +1.50 representing possible ceiling or floor effects, it was omitted from the scale (12, 19). Of the original 42 items, 14 were deleted because of low internal consistency with the other items or redundancy within a subscale. This resulted in a scale with five domains (general, eating, speech, cosmetic, and social/family life) (Figure 1).

The original version of this rating scale was written in Japanese, which was then translated into English by two professional native English and Japanese translators (6, 25) according to the standard protocol for back-translation (26). The final English version of the OMDRS is shown in the Appendix.

### Reliability

The internal consistency determined by pair-wise correlations among all items in the rating scale in all combinations of possible pairs of the subscales was quantified using Cronbach's alpha (12, 23). A minimum alpha value of 0.7 was considered as the cutoff value. To evaluate the severity of each subtype of oromandibular dystonia, four of the six items (A–F) were selected according to the subtype (jaw closing dystonia: A, D, E, and F; tongue dystonia: B, C, D, and E; jaw opening dystonia: B, C, D, and E; jaw deviation [protrusion] dystonia: A, B, E, and F; and lip dystonia: B, D, E, and F; Appendix).

To confirm test-retest reliability, scores on the OMDRS were compared in 52 patients (34 women and 18 men) at baseline and after 2 weeks by intraclass correlation coefficients (12).

The inter-rater reliability for the severity scale was assessed by five movement disorders experts who rated 15 video recordings according to a standardized protocol (Table 1). The experts did not know the patients who were recorded in the videos. The videos included three cases for each of the five subtypes. Inter-rater reliability was evaluated by kappa statistics and interclass correlation coefficients (12).

Three residents who had no expertise in oromandibular dystonia underwent brief training. They rated motor severity in patients using video tapes (Table 1) after a brief explanation of the OMDRS. Motor severity was rated again after 3 months. Reliability was assessed using kappa statistics and the interclass correlation coefficient (12).

### Validity

Content validity was indicated by relevant patient-generated aspects from the initial structured interviews. The Short Form Health Survey (SF-36) (25) and OMDQ-25 (11) were used to evaluate convergent and discriminant validity. The correlation between the total and subscales of OMDRS as well as the variables of these two scales were analyzed using Spearman's rank correlation. Considering the SF-36 scores in a direction different from the OMDRS, the SF-36 scores were converted accordingly.

Since the clinimetric properties CDIP-58 has already been validated (24), reliability and validity assessments were not conducted on the scale.

### Sensitivity to Change

Ninety-two *de novo* patients (59 women and 33 men) were assessed 4 weeks after the first injection of botulinum toxin (Botox®, Allergan; Irvine, CA) to evaluate the capability of OMDRS to detect therapeutic changes. Botulinum toxin therapy was performed as previously described (8, 27, 28). OMDRS scores were statistically compared between baseline and 4 weeks after botulinum toxin therapy.

### Statistical Analyses

All statistical analyses were performed using the statistical software package Statistical Package for the Social Sciences (SPSS) for Windows version 24.0 (SPSS Japan; Tokyo, Japan). The null hypothesis was rejected at the 5% level (*p* < 0.05).

### Patients

**Figure 2:**
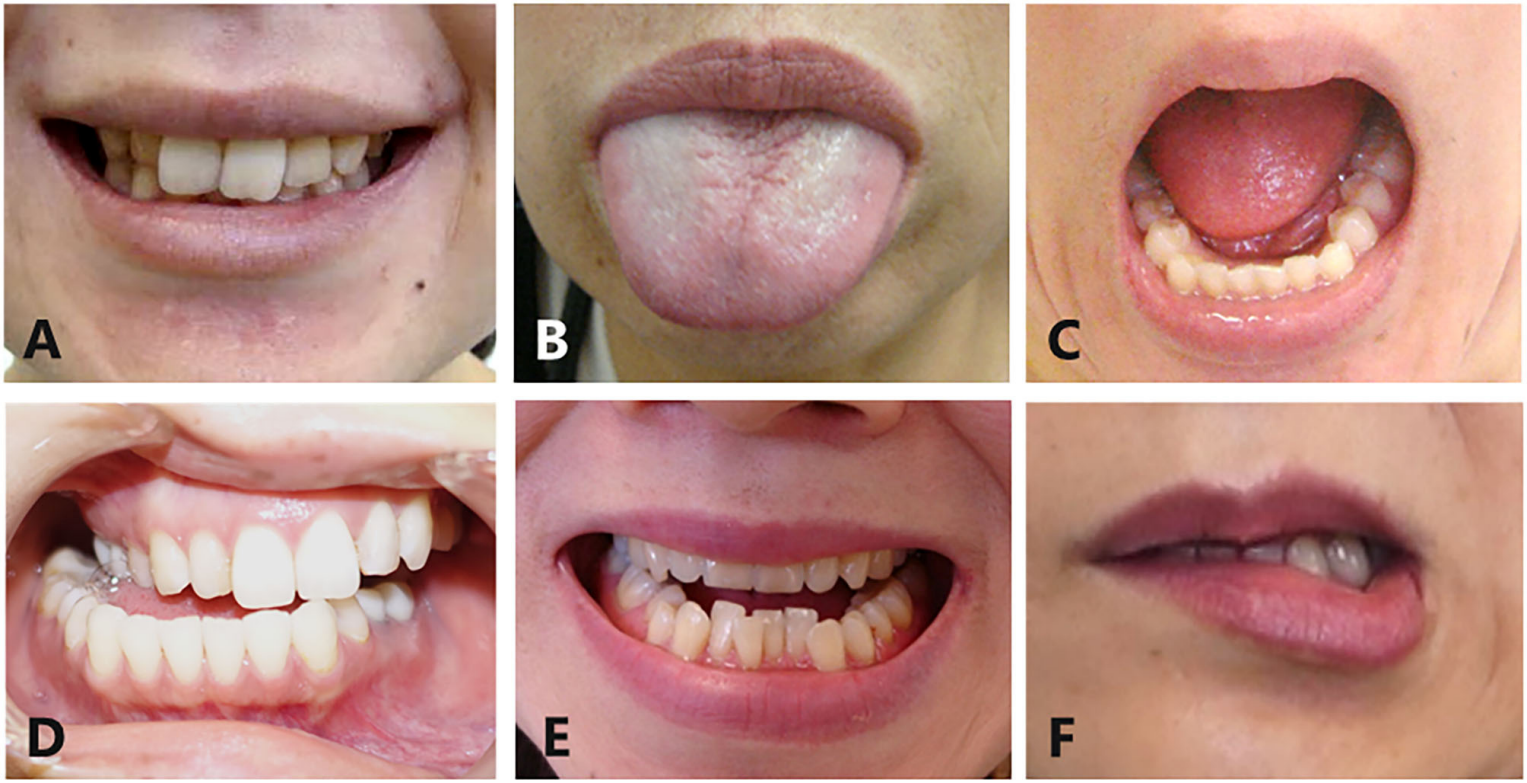
Subtypes of oromandibular dystonia. Oromandibular dystonia includes jaw closing dystonia **(A)**, tongue dystonia **(B)**, jaw opening dystonia **(C)**, jaw deviation dystonia **(D)**, jaw protrusion dystonia **(E)**, and lip dystonia **(F)**.

Oromandibular dystonia was diagnosed based on the characteristic clinical features of focal dystonia, such as stereotypy, task specificity, sensory tricks, overflow phenomenon, morning benefit, co-contraction, and electromyographic findings, as described previously (2, 6, 8). All patients involved in this study provided written informed consent after receiving a full explanation of the planned treatment. Patients were excluded if they had generalized dystonia or significant dystonia in other body regions as these patients were unlikely to have been able to precisely rate symptoms for oromandibular dystonia in isolation from other dystonic symptoms. Oromandibular dystonia includes jaw closing dystonia, jaw opening dystonia, tongue dystonia, jaw deviation dystonia, jaw protrusion dystonia, and lip dystonia (Figure 2). The patients were subdivided into five groups according to the six subtypes of oromandibular dystonia (jaw closing, tongue, jaw opening, jaw deviation [protrusion], and lip dystonia) based on their main symptoms, despite the presence of a combination of abnormal movements in some cases (Table 2). If two or more subtypes coexisted in a patient, the patient was classified as having the most severe subtype. One hundred and fifty-eight patients (25.6%) had other types of dystonia, such as cervical dystonia (12.1%) or blepharospasm (8.1%) (Table 2). However, the symptoms of these types of dystonia were very mild, and their chief complaints were symptoms associated with oromandibular dystonia.

**Table 2:** Demographic characteristics for each subtype of oromandibular dystonia and the entire patient cohort.

	**Jaw closing**	**Tongue**	**Jaw opening**	**Jaw deviation**	**Lip**	**Total**
Number of patients [*N*]	338	124	68	53	35	618
Age (years) [mean (SD)]	54.2 (16.2)	46.6 (14.1)	50.0 (18.2)	53.4 (16.6)	48.3 (14.6)	51.7 (16.2)
Sex (women, men) [*N* (%)]	228 (67.5) 110 (32.5)	73 (58.9) 51 (41.9)	32 (47.1) 36 (52.9)	37 (69.8) 16 (30.2)	24 (68.6) 11 (31.4)	394 (63.8) 224 (36.2)
Duration of symptom	52.5 (71.8)	30.0 (35.4)	42.3 (84.1)	41.9 (48.3)	56.9 (58.1)	46.2 (65.5)
(months) [mean (SD)]						
**Other dystonia [*****N*** **(%)]**						
Cervical dystonia	46 (13.6)	6 (4.8)	13 (19.1)	5 (9.4)	5 (14.3)	75 (12.1)
Blepharospasm	25 (7.4)	4 (3.2)	5 (7.4)	5 (9.4)	11 (31.4)	50 (8.1)
Writer's cramp	4 (1.2)	3 (2.4)	6 (8.9)	0	1 (2.9)	14 (2.3)
Lower limb	3 (0.9)	2 (1.6)	2 (2.9)	0	0	7 (1.1)
Upper limb	2 (0.6)	2 (1.6)	1 (1.5)	1 (1.9)	0	6 (1.0)
Spasmodic dysphonia	2 (0.6)	1 (0.8)	0	0	1 (2.9)	4 (0.6)
Embouchure dystonia	0	1 (0.8)	0	0	1 (2.9)	2 (0.3)

Six hundred and eighteen patients (394 women and 224 men; mean age, 51.7 ± 16.2 years) with oromandibular dystonia were evaluated using the OMDRS (Table 2). Before the rating, the maximum occlusal force, maximum mouth opening, protrusion, and lateral movements as well as protrusion or deviation of the tongue or lip were measured according to the subtype. The maximum occlusal force was measured bilaterally on the molars three times using an occlusal force meter (GM10, Nagano Keiki Co.; Tokyo, Japan). The patient was requested to speak or chew according to the video examination protocol to induced dystonia (Table 1), and the subsequent deviation was rated. Regarding involuntary mouth closing, the patient was requested to close the mouth maximally and forcefully. Subsequently, the patient was asked the following question: “What is the percent force exerted when you close mouth involuntarily compared to the maximum bite force you have just tried?” (Appendix).

Patients were interviewed to rate the severity, disability, and pain subscales. Subsequently, the questionnaire and subscales of CDIP-58 were administered to patients. The Japanese version of the OMDRS, SF-36, and OMDQ-25 were used on 602 Japanese patients, whereas 16 international patients completed the English version of the OMDRS and the original versions of the SF-36 and OMDQ-25.

This study was conducted in accordance with the Declaration of Helsinki under the approval of the institutional review board and ethics committee of Kyoto Medical Center (No. 09-37).

## Results

A total of 618 patients with oromandibular dystonia were evaluated using the OMDRS (Table 2). Of the 618 patients, 551 (89.2%) were newly diagnosed (*de novo*) in our department and had never received botulinum toxin therapy. Descriptive data from the OMDRS of all patients are shown in Table 2. None of the patients achieved theoretical minimum or maximum score values.

### Reliability

The overall OMDRS had a high level of internal consistency as measured by Cronbach's alpha (0.95). The internal consistencies for the subscales were satisfactory to excellent (Cronbach's alpha, 0.72–0.94). The overall Cronbach's alpha for the severity subscale was barely acceptable (0.50). The Cronbach's alpha for the five subtypes ranged from 0.72 to 0.80 (Table 3). The overall Cronbach's alpha for the four subscales of the CDIP-58 was 0.96.

**Table 3:** Descriptive statistics and internal consistency of the oromandibular dystonia rating scale.

**OMDRS subscale (no. of items)**	**Jaw closing (*N* = 338) mean (SD)**	**Tongue (*N* = 124) mean (SD)**	**Jaw opening (*N* = 68) mean (SD)**	**Jaw deviation (*N* = 53) mean (SD)**	**Lip (*N* = 35) mean (SD)**	**Total (*N* = 618) mean (SD)**	**Internal consistency (Cronbach's α)**
**Examiner-rated scale**							
Severity (4)	7.2 (2.7)	7.5 (3.0)	7.9 (3.8)	6.6 (2.4)	5.6 (2.3)	7.3 (2.9)	Jaw closing: 0.80 Tongue: 0.74 Jaw opening: 0.76 Jaw deviation: 0.72 Lip: 0.73
Disability (6)	9.8 (5.9)	9.7 (4.7)	14.2 (7.3)	8.4 (4.1)	9.0 (5.9)	10.1 (5.8)	0.78
Pain (5)	11.4 (10.9)	4.4 (7.4)	10.3 (11.9)	7.4 (8.8)	5.3 (9.8)	9.0 (10.4)	0.91
**Patient-rated questionnaire**							
General (5)	14.2 (4.4)	14.9 (3.3)	14.5 (4.1)	14.7 (4.2)	13.8 (3.9)	14.4 (4.1)	0.79
Eating (7)	11.0 (7.4)	7.7 (7.5)	14.4 (7.2)	10.0 (7.2)	10.0 (8.2)	10.5 (7.6)	0.86
Speech (4)	7.5 (4.6)	11.7 (4.1)	9.4 (4.3)	9.1 (4.1)	10.9 (4.7)	9.0 (4.8)	0.90
Cosmetic (7)	11.8 (7.8)	14.0 (7.3)	16.9 (7.3)	16.5 (7.5)	16.4 (8.7)	13.5 (7.9)	0.87
Social/family life (5)	8.3 (5.7)	10.0 (5.0)	11.1 (4.9)	9.4 (5.3)	9.2 (4.8)	9.2 (5.5)	0.83
**CDIP-58**							
Sleep (4)	5.0 (5.0)	3.9 (5.0)	6.1 (5.4)	3.5 (4.8)	3.3 (4.1)	4.7 (5.0)	0.94
Annoyance (8)	15.7 (7.3)	17.9 (7.5)	19.2 (7.9)	18.2 (7.0)	14.2 (6.9)	16.8 (7.4)	0.86
Mood (7)	15.9 (7.9)	17.6 (7.0)	17.4 (8.3)	18.1 (7.2)	13.4 (8.0)	16.6 (7.7)	0.91
Psychosocial functioning (10)	14.6 (10.4)	17.5 (9.5)	18.6 (9.4)	15.0 (9.5)	17.0 (9.7)	15.8 (10.0)	0.90
**Total OMDRS**	131.2 (53.4)	135.5 (42.7)	156.8 (51.6)	134.9 (46.3)	126.0 (49.7)	135.3 (50.4)	0.95

The test-retest reliability showed a significant correlation efficiency (*p* < 0.001). Intraclass correlation coefficients were 0.9 or higher for all domains.

All items revealed acceptable inter-rater reliability (kappa > 0.4, interclass correlation coefficient > 0.6).

Repeated ratings of videotapes revealed acceptable intra-rater reliability for all items (kappa > 0.76, interclass correlation coefficient > 0.86).

### Validity

The assessment of convergent validity revealed significant (*p* < 0.001) correlations between the final version of the OMDRS and the other two scales (SF-36, *r* = 0.389; OMDQ-25, *r* = 0.787). Significant correlations between the subscales of the OMDRS, OMDQ-25, and SF-36 are shown in Table 4. A high correlation was observed between the OMDRS and OMDQ-25 in some subscales (Table 4).

**Table 4:** Correlations of the subscales of the oromandibular dystonia rating scale, Short Form-36 Health Survey (SF-36), and Oromandibular Dystonia Questionnaire (OMDQ-25).

**OMDRS subscale**	**SF-36**			**OMDQ-25**		
	**subscale**	***r***	***p***	**subscale**	***r***	***p***
Severity	–	–	–	–	–	–
Disability	–	–	–	Eating dysfunction	0.564	0.001
Pain	Bodily pain	0.357	0.001	Eating dysfunction	0.468	0.001
General	Role emotional	0.342	0.001	General	0.734	0.001
Eating	Physical functioning	0.320	0.001	Eating dysfunction	0.821	0.001
Speech	–	–	–	Speech	0.832	0.001
Cosmetic	–	–	–	Cosmetic	0.755	0.001
Social/family life	Social functioning	0.390	0.001	Psychosocial	0.760	0.001
Sleep	Role emotional	0.351	0.001	Psychosocial	0.496	0.001
Annoyance	Social functioning	0.370	0.001	Psychosocial	0.650	0.001
Psychosocial functioning	Role physical	0.306	0.001	Psychosocial	0.668	0.001

### Sensitivity to Change

Comparisons of total OMDRS scores before and after botulinum toxin therapy in 92 patients revealed a significant (*p* < 0.001) decrease after treatment (135.3 ± 50.4 vs. 55.2 ± 33.1). Patients improved significantly (*p* < 0.001) from baseline to 4 weeks after botulinum toxin therapy in all OMDRS subscales. The effect sizes for the total score and all subscores were >0.75.

## Discussion

The present study is the first to assess the reliability and validity of a comprehensive disease-specific rating scale for patients with oromandibular dystonia (OMDRS).

The Toronto Western Spasmodic Torticollis Scale (TWSTRS) is the most widely utilized rating scale for cervical dystonia. TWSTRS-2 includes assessments of motor severity, pain, and disability (17). TWSTRS-PSYCH includes items from established psychological rating scales (17). The CDIP-58 is a self-administered scale with eight subscales for evaluating head and neck symptoms, pain and discomfort, sleep, upper-limb activities, walking, annoyance, mood, and psychosocial functioning (24). The CDQ-24 is a patient-rated health-related quality of life measure for craniocervical dystonia (13). Comella et al. (17) reported the Comprehensive Cervical Dystonia Rating Scale (CCDRS), which includes a revision of the TWSTRS-2, TWSTRS-PSYCH, and CDIP-58. Merz et al. (11) developed and validated the OMDQ-25, which comprises five subscales (general, psychosocial, cosmesis, speech, and eating dysfunction). The scale was the first instrument to measure health-related quality of life for patients with oromandibular dystonia. However, the Movement Disorders Society Task Force on dystonia rating scales did not recommend the OMDQ-25, but merely suggested it, because it was only used by the original developers and not by other researchers (14).

The development and validation of rating scales for dystonia (13, 17–19) and Parkinson's disease (19–21) have been completed through multicenter studies at several university hospitals or movement disorder centers with interdisciplinary teams in dystonia, neurology, psychiatry, clinimetrics, and biostatics. However, since there are no hospitals or departments that specialize in oromandibular dystonia in Japan or other countries, the development and validation of the OMDRS were conducted by an oromandibular dystonia specialist. All study patients were diagnosed, treated, evaluated, and followed-up by the same expert to ensure uniformity of results. The OMDQ-25 is a concise patient-rated 25-item questionnaire (11). On the other hand, OMDRS includes 15-item examiner-rated scale and 57-item patient-administered questionnaire to objectively and comprehensively evaluate full spectrum of oromandibular dystonia. The OMDRS can be useful for more precisely evaluating disease severity and post-treatment changes of each subtype for both clinical and research purposes.

Although we attempted to elucidate the pathophysiology of oromandibular dystonia using several neuroimaging techniques (29–33), its etiology remains unclear. The symptoms of oromandibular dystonia exhibit extremely large individual differences from patient to patient. For instance, jaw closing, jaw opening, and tongue dystonia are completely different in terms of clinical features, muscles with dystonic contracture, and direction of abnormal movements. The variety of symptoms may be significantly higher in patients with cervical dystonia or blepharospasm. Therefore, the severity subscale should be examined according to subtypes of oromandibular dystonia. In a preliminary version of the rating scale, the author attempted to evaluate disease severity using the same subscales for all types of oromandibular dystonia. However, if the items are negatively associated within a subscale, the subscale cannot reach sufficient internal consistency as assessed by Cronbach's alpha (12). Comella et al. deleted such items from the motor severity subscales when they developed the CCDRS (19). In contrast, in this study, only the severity subscale (four items) was assigned five patterns according to the subtypes of oromandibular dystonia (jaw closing, tongue, jaw opening, jaw deviation [protrusion], and lip dystonia) (Appendix). While this may be complicated, it enables a more precise evaluation of the severities of each subtype.

The study population in this report, which particularly included patients with isolated oromandibular dystonia, may significantly differ from that in neurological departments, where oromandibular dystonia is secondary to neurological diseases in most patients. Patients with secondary and generalized dystonia and significant coexisting neurological conditions were excluded from the analysis to precisely rate symptoms of oromandibular dystonia from other symptoms. Oromandibular dystonia is often misdiagnosed as temporomandibular joint disorder, psychogenic disorders, bruxism, or conditions of unknown etiology (6, 34, 35). Although the patients had consulted an average of 3.9 departments or hospitals over a long period before they visited our clinic, only 12.5% had been diagnosed with or were suspected of having dystonia (35). Oromandibular dystonia is a blind spot between medicine and dentistry; thus, patients tend to consult dental and medical professionals who may not necessarily have the appropriate experience for treating this disease. After the author launched a website for involuntary movements (https://sites.google.com/site/oromandibulardystoniaenglish/), many misdiagnosed or unrecognized patients who had already abandoned treatment or further consultation visited our clinic (35). The prevalence of oromandibular dystonia has been estimated at 68.9 per million (36). Nevertheless, the real prevalence of oromandibular dystonia must be significantly higher than previously estimated.

Differentially diagnosed patients have been treated with botulinum toxin injection (8, 27, 28, 37), muscle afferent block (2, 3), sensory trick splint (38), and coronoidotomy (4, 5, 39). Although an early double-blinded placebo-controlled study (40) using a suggested scale has been reported, the evidence levels of studies on oromandibular dystonia are not always satisfactory compared with that of those on other types of dystonia. The strength of evidence is derived not only from the quality of the study design but also from the use of a validated disease-specific rating scale (15). The rating scale described here may be useful for evidence-based clinical studies on oromandibular dystonia. If clinicians or researchers gain interest in such studies, it might result in clinical trials with higher levels of evidence comparable to those in other types of focal dystonia.

## Conclusion

The OMDRS can be useful for the comprehensive evaluation of disease severity, disability, psychosocial functioning, and impact on the quality of life as well as therapeutic changes in patients with oromandibular dystonia.

## Data Availability Statement

All datasets generated for this study are included in the article/Supplementary Material.

## Ethics Statement

This study was performed in accordance with the Declaration of Helsinki under the approval of the institutional review board and ethics committee of Kyoto Medical Center (09-37). All patients in this study provided written, informed consent after receiving a full explanation of the planned treatment.

## Author Contributions

KY diagnosed and treated all patients, analyzed the results, and wrote the manuscript.

## Conflict of Interest

The author declares that the research was conducted in the absence of any commercial or financial relationships that could be construed as a potential conflict of interest.
